# Abstract of the Proceedings of the Buffalo Medical Association

**Published:** 1879-02

**Authors:** Joseph Fowler

**Affiliations:** Secretary


*ART. II.—Abstract of the Proceedings of the Buffalo Medical Association, Dec. 3d,* 1878. Reported by JOSEPH FOWLER, M. D., Secretary.

The Association was called to order by the President, Dr. LOTHROP.

There was a fair attendance of members.

The minutes of the last meeting were read and adopted, Dr. P. H. STRONG, the Essayist appointed for the occasion, read the following paper, which, upon motion, was referred to the BUFFALO MEDICAL AND SURGICAL JOURNAL for publication.

*Mr. President and Gentlemen of the Association:*

When I promised your committee that I would furnish a paper to serve as the basis for an evening's discussion, I had in mind certain clinical observations and the deductions therefrom, which really might better come up as matters of talk than otherwise. But as I have such a poor faculty of thought and expression when upon my feet, I have concluded that it would be more satisfactory and profitable all round, to indite and thus present them for consideration.

You do not need to be reminded that the requirements of the period of Gestation, and preeminently those of the subsequent accouchment and the puerperal condition, make very heavy drafts upon our time, and not less upon our mental resources and our skill, while engaged in active general practice. In the exceptionally healthy locality in which our lot is cast, it may not be extravagant perhaps to say that the most of our time is thus occupied—certainly it is within the mark to say, that no class of professional duties are so absorbing and anxious to mind, so taxing to patience or so wearying and exhausting to the entire vital energies, physical and mental, as is that incident to and connected with the Lying-in-Room, so called. Diseases may come and diseases may go, (in fact *do* come and go according to observance or non-observance of well known sanitary and dietary laws,) but child bearing and its perils, go on forever.

But without farther preamble let us come to the specific theme upon which I am expected to discourse to you for the evening, this viz: Certain points pertaining to Puerperal Fever and the Puerperal condition.

I have not the temerity, Gentlemen, to propose to discuss within the limits of twenty or thirty minutes so vast a subject as is generally included under the term Puerperal Fever in its entirety. A good sized library might be gathered upon this one subject. My limitations do not admit of presenting to you its histology, morbid anatomy, not much as to its Pathology or essential nature, still less concerning its treatment. But I have reached certain convictions concerning what may be termed sporadic Puerperal Fever, especially as to its causation and Prophylaxis, that being the fruit of some thirty-five years of observation and experience, of course have controlling weight with me. To you, whether you should consider them wise or otherwise, they may serve as the hinge upon which the evenings discussion may not unprofitably turn.

As you are aware, the term Puerperal Fever is used to cover and include four or five (or more) somewhat distinct affections and Pathological conditions, viz: Puerperal Metritis, (pure and simple)

Puerperal Peritonitis, pure and simple, Puerperal Metre-Peritonetis, Uterine Phlebitis, Uterine Pyemia, and perhaps others, all having points of difference, but all alike deriving their significance and gravity from the Puerperal condition. Now, what is that condition. As practical men, you do not require to be told that it derives nearly all, if not its entire importance from the condition of that portion of the uterus covered by the now detached and removed Placenta. The torn and bleeding uterine vessels are closed by coagula and the contraction of the organ, and when contracted to its minimum we anticipate and generally experience no farther morbid results. It is a physiological condition adequately provided for by simple natural forces and processes. Like the wound with its surfaces brought in opposition it may be said to heal by first intention.

But how different with the uterus inadequately contracted. The severed vessels without the contracting pressure upon them are left patulous and bleeding more or less, and being unclosed or imperfectly closed by the clot formation, besides the imminent peril from hemorrhage it requires but a few hours continuance of this state to develope the suppuration process which is inevitable by the first intention. Moreover, the enlarged vessels left undiminished or inadequately diminished, even when closed by clots are liable and ready to assume morbid conditions from the presence of coagula in a preternatural amount. The clot itself, being excessive proving by its disintegration and decomposition, a morbific element by developing Septicemia. So that we may easily have super-added to the product of the suppuration process, or possible *Pyemia*—blood contained in the ruptured and uncontracted vessels in excess of the requirements of the reparation process, and therefore left to undergo decomposition and putrefaction, and thus possible *Septicemia.* Add to this coagula unexpelled from the uterine cavity, (we all know how soon the putrefaction change sets in to blood in this situation,) and we need seek no farther for possible, if not probable causes of Puerperal Fever in some one of its forms. Indeed I have often wondered not, why and how it could occur, but how any woman with uncontracted or even poorly contracted uterus after child-birth could escape. It is now, I believe, generally conceded by our best Gynecologists that the disease is not infrequently communicated by the accoucheur after days or even weeks have elapsed since his attendance upon a case of the disease, and despite the utmost care on his part, by way of ablutions, disinfectants and change of apparel.

Now without the slightest attempt to solve the mystery of the means or mode of communication, it would seem to be demonstrated not only that the material of infection is exceptionally intangible and virulent, but that the fresh puerperal condition imparts to the uterus and tissues involved, a wonderful susceptibility to the toxical effects of this special infection.

The scope of the present paper utterly precludes the consideration of the various forms of the disease and their differentiation except to express the conviction, that Puerperal Peritonitis, pure and simple, is a myth, and that what is so called and regarded, is invariably preceded or accompanied (generally the former) by Metritis. I account for the error by the well known disparity existing between the Peritoneum and the Uterine tissues as to their respective endowment with sensitive-nerves—the slightest inflammation of the former causing more or less intense suffering upon pressure, while much grosser inflammation of the uterus may be wholly painless, and so undetected.

From the foregoing consideration it would seem not an unfair deduction, that we have in the case of the inadequately contracted uterus, and from the consequent and inevitable suppurative process set up on one hand, and from the superfluous blood clots retained in the morbidly dilated uncontracting blood vessels, as also in the cavity of the uterus on the other hand, and thus possible if not unavoidable Pyemia and Septicemia absorption, I repeat, that in these conditions we have sufficient, and more than sufficient cause to account for the Pathological phenomena existing in Puerperal Fever, and every phase and form of it. And the point I would make in this connection is, that every case of sporadic Puerperal Fever is traceable primarily to inadequately contracted uterus, and the resulting purulent or septic absorption, one or both. It is utterly immaterial to my mind, whether it assumes the form of Metritis, Metro-Peritonitis, Uterine Phlebitis, Uterine Pyemia or what not, they are all alike traceable to this source. I fully believe that all the symptomatic as also all the post-mortem phenomena are explicable upon this view of the causation of this formidable disease.

Now, if this position is correct, and I believe it incontrovertible, the corrollary in its practical suggestions is most obvious, this, viz: that to secure after delivery, the contraction of the uterus to its minimum is of vast, and often, of vital importance. Can I be mistaken in the belief that precisely this point in practice, is too often overlooked?

It is said to be a good rule, though not always fair, perhaps, "to judge others by oneself," but it is certainly more likely to be so when it involves self-delinquency and confession. Confession is not only good for the soul, according to adage, but it may also be profitable to others by mirroring and recalling facts and events in their own experience. The present essayist then would frankly confess, that he had been in practice a score of years or more, ere he adequately apprehended the full significance, the vast importance of securing uterine contraction, post partum, to its almost minimum before he left the room of accouchment.

The authorities were sufficiently explicit as to details of duty up to the moment of delivery, whether natural or artificial—then as to removal of the placenta, and if undue hemorrhage occurred as to arresting it by various means, among which uterine contraction was of importance. No external hemorrhage occurring, and the patient being every way comfortable, it was generally thought and taught that the requirements of the case were met, and the patient could be safely consigned to the attendant nurse. If there was reason to fear hemorrhage, it was enjoined to remain with patient until the danger seemed to be past. If flooding was present or apprehended, the size of the uterus should attract attention and effort at its reduction,—otherwise it was of small account. Meddlesome midwifery we were told, was bad and must not be indulged in.

Such, briefly, were the inculcations of the authorities in the days of my pupilage, and such, I believe it largely remains to this day. Such certainly was my own mode of procedure for the first twenty years, or more of my practice. And it is due to truth and fairness to add, that such procedure in by far the most of cases, meets the requirements, and we have high authority for saying that, "All's well that ends well."

During the period specified, I can enumerate but very few cases of sporadic Puerperal Fever, probably not two per centum of cases of confinement. Cases which amounted, in one way or another, to slow and poor recoveries, and which I have known, were due to poorly contracted uterus, as well as my Puerperal Fever cases, were more numerous. Still I suppose that my experience during that period was about that of the average of practitioners. But be this as it may, I have reached the decided conviction, that, with the exception of drawbacks from sore nipples and mammary abscess nearly all, if not all the untoward recoveries from child-birth are traceable more or less directly, (generally more) to imperfect contraction, and as pertains to sporadic Puerperal Fever, (the special objective point at which this paper is aiming,) I soberly believe, that it is uniformly thus traceable.

The question naturally arises, can anything be done to obviate or avert this affection with other allied puerperal conditions only less grave and dangerous? It is my conviction, that much, if not everything can be thus done. And with a brief exposition of the means and methods to this end, I will relieve your patience and submit the matter for your consideration.

Ordinarily of course, unaided nature is equal to the requirements. In the considerable proportion of cases that are exceptions to the rule, what can be done. In the case of undue hemorrhage, post partum, we meet the exegency by prompt efforts and well known methods to secure uterine contraction—among which are external pressure, and firmly grasping and squeezing the faultily contracted organ. This failing, according to the urgency of the case, we introduce the hand, remove the clots if present, make pressure upon the internal surface of the uterus and more especially upon the placental site and bleeding vessels. This failing the hand may be chilled in cold water and reintroduced wet, and the pressure maintained if necessary, the uterine walls pressed firmly between the two hands, the one external and the other on its internal surface. By one or the other means the organ contracts and the hemorrhage is arrested. In cases of undue hemorrhage we feel the necessity of protracting our stay and keeping the patient under close surveillance until all danger of flooding has ceased. It may be advisable also to superadd to our manual means the free use of ergot, with other potencies which I will not stop to indicate. But in a certain proportion of cases there is no excessive external hemorrhage, and yet the organ remains of undue size and noncontractile. It often occurs in this case, that while there is no external flooding, the blood-flow continues too profuse, but is so gradual as not to escape externally, but forms coagula which quite distend the flaccid uterus. Indeed it is not very uncommon with me, as I suppose is the case with you, Gentlemen, to have the organ within a half hour after delivery and after having attained great reduction in size to become again considerably distended by accumulating coagula, and all this with slight external hemorrhage, and often with no alarming symptoms by way of exhaustion of my patient.

Now what is good practice in such a case. We find on pressing the hand upon it that the organ either remains undesirably large, or seems to expand after having contracted, either from coagula or otherwise, and yet no alarming external flooding, and no untoward symptoms other than this excessive size. I believe that the ordinary rule in practice is, after brief efforts, to cease further attention to the organ, and after remaining twenty or thirty minutes or more, and no flooding externally occurring, and upon giving strict injunctions to the nurse for contingencies, to trust the case to the recuperative forces of nature. The patient, more or less exhausted from more or less protracted suffering, desires to be let alone, or to have no painful methods, extra or intra-uterine resorted to. There seems to be no indication, certainly no urgent indication for further interference.

We call to mind, it may be, other similar cases, where everything resulted favorably, and it must be confessed that the majority of such cases do thus result. The distended uterus, whether from coagula or not, is generally equal to the exigency, and more or less gradually contracts, and if clots are present they are for most part, sooner or later expelled, and the uterus assumes its wonted place and nearly its minimum size. The patient may have a somewhat protracted getting up, but generally recovers with nothing, at least nothing so serious as Puerperal Fever; and again, All's well, that ends well," apparently.

I have to confess, that, however it may be with others, this, for many of the earlier years of my practice, was my mode of procedure in the Lying-in-Room. A very small per centage of cases of Puerperal Fever, and a somewhat larger proportion of annoying tedious recoveries from one drawback or another was the result. A careful study of my untoward cases, at length convinced me that this mode of practice could be improved upon, and in accordance with this view, for the past ten or fifteen years, I have regarded my duty to my patient not discharged without making urgent and presistent efforts to reduce the dimensions of the uterus to its utmost minimum before leaving the accouchment chamber.

Whether the unduly disturbed organ remains flaccid or of a ball-like hardness, even in the absence of excessive flooding, I do not now remit efforts at its reduction by firm sustained pressure, and, that failing, I am in the habit of grasping the organ with both hands, one on either side, and squeezing it with such force that the clots which may retard its contraction are pressed out of it. But, all this failing to effect its reaction, one hand is introduced into the uterus to scoop out the coagula and press upon its internal surface, while the other maintains the pressure or grasping external. This is done at any time within half an hour or more after removal of placenta. Once done is usually sufficient and insures contraction, but failing of that, and the coagula reforming, they are again to be dislodged by the same procedure. By determined persistence in this course, I do not recall the case of late years in which I have failed of the desirable contraction.

These practical suggestions are made, with no thought or design of instructing you, who may be said to belong to the veteran corps. They are thrown out as hints to guide my medical brethren who are as yet wholly or largely inexperienced in this special field of practice. To such I would add the caution not to give up or suspend these urgent efforts at reduction at the request or even entreaty of the patient to be let alone or her more or less loud outcries at the trifling pain incidental to the extra or intra manipulations. It must be confessed that it often requires not a little nerve in the absence of apparent indication or necessity for it in the mind of the patient, and following so closely, it may be distressing and exhausting labor to persist in the use of the means necessary for securing adequate contraction; but none the less important, as it strikes me, of effecting just that. I have found it usually sufficient to represent it as conducive to their future well-being by forestalling possible drawback to a speedy and solid recovery.

In conclusion, allow me to add that it comports neither with a considerate regard to your powers of endurance, nor with the design and scope of this paper to illustrate the points made in it by representing individual cases, which might be done indefinitely. It will suffice for my present purpose to give the net result by stating that, if I except the case of a lady pregnant and confined for the first time when over 40 years of age and delivered by forceps with extreme difficulty, I have had, after 10 years of practice upon the views and principles and by the methods I have here endeavored to describe and advocate, nothing worthy of dignifying by the term puerperal fever in any of its forms—nor any other puerperal accident that in the least imperilled life or caused any considerable anxiety. And if I except occasional sore nipples and mammary abscess, I have had scarcely a case that could fairly be said to be anything but a rapid and solid recovery from childbirth. I can attribute it to nothing so much, if at all, as to my carrying into effect the rule I have adopted, not to leave accouchment room without having made urgent and persistent efforts at reducing the post-partum uterus to its minimum whenever unassisted nature is unequal to the task. I speak to wise men—judge ye!

Dr. White. The paper not only brought up one subject, but a number. Dr. Strong's remarks appear to have been confined to the sporadic form of puerperal fever. I think it wise to discriminate between the tissues affected, and as important to do so early as in pulmonary disease.

I do not think the uncontracted uterus the sole cause of the affection, but have always appreciated the necessity of securing firm contraction of the organ before leaving the lying-in-chamber.

The books all contain this advice, and I have taught it for more than thirty years, and never omit to charge my students to remain at least an hour after the delivery of the child. Labor is a psycological condition and not a pathological one. The mother is aware of this, and knows perfectly well that under ordinary circumstances she must unaided by the physician give birth to her child.

She knows also that hemorrhage is liable to take place, and that there are other sources of danger. These are the reasons which cause her to send for a physician, and he utterly fails to fulfill his contract with her should he retire before all danger is past.

I think it wicked to leave the bed-side before the uterus is firmly contracted. I rarely consider it necessary to introduce the hand into the uterus to remove clots. I am not prepared to endorse Dr. Strong's views as to the cause of the sporadic form of puerpural fever.

The great danger to be apprehended from an uncontracted uterus is hemorrhage.

No poison is more easily transmitted than that of puerperal fever. It is communicated from one patient to another through the attending physician, the wet or monthly nurse, and the visitations of officious friends.

I believe with Brundell. I would rather a friend be confined in a stable, unattended, than in the fairest apartments surrounded by every comfort and exposed to the poison of the disease.

Puerperal fever of the most dangerous type may be contracted from inflammatory erysipelas or surgical fever. These two diseases begin together, progress together, and disappear at the same time. They are conveyable diseases. Those engaged in making a post-mortem examination or dissecting should not attend a case of labor on the same day. The poison is liable to be absorbed from the uterine surface and produce puerperal fever.

I have purposely avoided discussing the subject of post-partum hemorrhage. It is a very important one, and I trust the association will take it up at some future meeting.

Dr. Miner. I do not think the cause of puerperal fever has been quite satisfactorily explained. It is in epidemic form a most fearful disease and alarmingly fatal; disinfecting agents have apparently no controlling influence over the spread of the disease when caused by contagion.

Kneading of the uterus after delivery to secure firm contraction of the organ may possiby be efficacious; but should there follow inflammation of any of the tissues the question might arise, was not this the cause of the disease; am disposed to doubt the value of the kneading process in producing contraction of the womb. Blood coagula should be removed from the neck of the womb when attended with hemorrhage, and introduction of the hand into the womb in cases of sudden and violent flooding often seems to promote uterine contraction. Have often seen hemorrhage arrested by removing clots from the uterine neck. This I have found sufficient in nearly all cases.

I am not in the habit of remaining an hour with my patient after delivery, neither do I regard it necessary only in exceptional cases, and have never yet had occasion to regret an earlier absence.

I never could convince myself that we gained anything by binding the uterus down, as is usually done. I regard ergot as a valuable agent to produce uterine contraction, where the womb will not contract of its own accord, but I prescribe it as little as possible.

Dr. Strong said he meant it to be understood that the introduction of the hand in the uterus was rarely demanded and then only as a last resort.

He brought up the subject of causation to show the extreme unsusceptibility of the tissue to the action of the morbific influence, and urged the importance of securing firm, permanent contraction immediately after delivery; one of its objects being to abbreviate the stage of suppuration.

Was glad to hear Dr. Miner say he believed practitioners were in the habit of leaving the lying-in chamber in twenty minutes to half an hour, but was astonished that a veteran physician should be distrustful of external manipulation and tight bandaging.

Dr. Johnson. I arise to thank Dr. Strong for his comprehensive and valuable paper. So much has already been said that it is hardly proper for me to occupy the time of the association. I have but very seldom met with cases where it was necessary or proper to introduce the hand to produce uterine contraction, and think we should be slow to adopt the procedure recommended in the paper. I fully agree with Dr. Strong in the propriety of making firm external pressure and manipulations. I have more confidence in ergot than some members of the profession seem to have and believe that if it is properly given just before or immediately after the expulsion of the child sufficient contraction will almost invariably follow.

I know that Prof. White has for a long time taught that the accoucheur should not leave the patient in less than an hour after the completion of labor, but I have not always followed his teachings to the letter.

I frequently remain less than that time, and am governed by the patient's condition in each individual case. I do not believe it necessary for every woman to remain in bed nine days after confinement. It is just as safe for some women to leave the bed on the seventh or eight or tenth day as others on the twelfth or thirteenth. Do not believe in minutes or days, but am governed by the circumstances of each particular case.

Dr. Bartlett. The subject seems to have new interest because of its partial neglect.

Am glad to hear Dr. Strong attach so much importance to the practice of external pressure and manipulation after the expulsion of the child. I would go still further than this and rely on pressure and external manipulation to secure the delivery of the placenta. For ten years I have entirely relied on this plan and found it invariably successful.

Forced contractions on the cord or the introduction of the hand in the uterus was rude, uncalled for and liable to be attended with danger. It might induce *transmitted* hemorrhage and leave the surface more susceptible to the absorption of the decomposed matter.

In a paper read before this association some time ago I urged the importance of removing the pillow from beneath the head near the close of labor, to get an increased supply of blood to the brain, recuperating nervous force.

I believe it quite essential to remain with my patient fully an hour after delivery of the child, if for no other reason than that of the safety of the child, and always assure myself that uterine contraction is good and the umbilical ligatrix secure.

The Doctor here alluded to his successful use of the sponge moistened with a weak solution of Ferri. Per. Sulph., with cord attached, and pressed to the fundus of the womb, in a remarkable case of post-partum hemorrhage.

Dr. Wetmore. It has been my practice to give ergot just before the child is born. I do not think ergot has any effect on an empty uterus. Can secure contraction by firmly grasping the uterus and making external manipulations. In some cases hardly anything will stop hemorrhage. Have found electricity to succeed where other means failed. I rarely introduce my hand into the womb. Clots will sometimes form where constant pressure is being made. In a severe case of hemorrhage I once secured perament contraction by introduciug a piece of ice about the size of a hen's egg in the uterus. It is my opinion puerperal disease is more prevalent in the country than in the city.

It was my misfortune to lose two cases at a time when I was engaged in dessecting and have since thought I communicated the disease to them.

Of remedial agents I place my main reliance on opium, whiskey, quinine and the application of manipulaion to the abdomen.

Scarlatina and diphtheria were reported as prevailing in the city.

On motion Dr. Strong's paper was referred to the editor of the Buffalo Medical and Surgical Journal for publication.

